# Impact of Improving Community-Based Access to Malaria Diagnosis and Treatment on Household Costs

**DOI:** 10.1093/cid/ciw623

**Published:** 2016-12-06

**Authors:** Joëlle Castellani, Jesca Nsungwa-Sabiiti, Borislava Mihaylova, IkeOluwapo O. Ajayi, Mohamadou Siribié, Chinenye Afonne, Andrew Balyeku, Luc Sermé, Armande K. Sanou, Benjamin S. Sombié, Alfred B. Tiono, Sodiomon B. Sirima, Vanessa Kabarungi, Catherine O. Falade, Josephine Kyaligonza, Silvia M. A. A. Evers, Aggie T. G. Paulus, Max Petzold, Jan Singlovic, Melba Gomes

**Affiliations:** 1 Department of Health Services Research, School for Public Health and Primary Care (CAPHRI), Maastricht University, The Netherlands; 2 Child Health Division, Ministry of Health, Kampala, Uganda; 3 Health Economics Research Centre, Nuffield Department of Population Health, University of Oxford, United Kingdom; 4 Groupe de Recherche Action en Santé, Ouagadougou, Burkina Faso; 5 Department of Epidemiology and Medical Statistics; 6 Epidemiology and Biostatistics Research Unit, Institute of Advanced Medical Research and Training; 7 Department of Pharmacology and Therapeutics, College of Medicine, University of Ibadan, Nigeria; 8 Centre for Applied Biostatistics, Occupational and Environmental Medicine, Sahlgrenska Academy, University of Gothenburg, Sweden; 9 UNICEF/UNDP/World Bank/WHO/Special Programme for Research & Training in Tropical Diseases, World Health Organization, Geneva, Switzerland

**Keywords:** CHW, economics, access, ACTs, malaria

## Abstract

***Background.*** Community health workers (CHWs) were trained in Burkina Faso, Nigeria, and Uganda to diagnose febrile children using malaria rapid diagnostic tests, and treat positive malaria cases with artemisinin-based combination therapy (ACT) and those who could not take oral medicines with rectal artesunate. We quantified the impact of this intervention on private household costs for childhood febrile illness.

***Methods.*** Households with recent febrile illness in a young child in previous 2 weeks were selected randomly before and during the intervention and data obtained on household costs for the illness episode. Household costs included consultation fees, registration costs, user fees, diagnosis, bed, drugs, food, and transport costs. Private household costs per episode before and during the intervention were compared. The intervention's impact on household costs per episode was calculated and projected to districtwide impacts on household costs.

***Results.*** Use of CHWs increased from 35% of illness episodes before the intervention to 50% during the intervention (*P* < .0001), and total household costs per episode decreased significantly in each country: from US Dollars (USD) $4.36 to USD $1.54 in Burkina Faso, from USD $3.90 to USD $2.04 in Nigeria, and from USD $4.46 to USD $1.42 in Uganda (all *P* < .0001). There was no difference in the time used by the child's caregiver to care for a sick child (59% before intervention vs 51% during intervention spent ≤2 days). Using the most recent population figures for each study district, we estimate that the intervention could save households a total of USD $29 965, USD $254 268, and USD $303 467, respectively, in the study districts in Burkina Faso, Nigeria, and Uganda.

***Conclusions.*** Improving access to malaria diagnostics and treatments in malaria-endemic areas substantially reduces private household costs. The key challenge is to develop and strengthen community human resources to deliver the intervention, and ensure adequate supplies of commodities and supervision. We demonstrate feasibility and benefit to populations living in difficult circumstances.

***Clinical Trials Registration.*** ISRCTN13858170.

It is estimated that approximately 214 million cases of malaria occurred in 2015, almost 90% in sub-Saharan Africa [1]. Children aged <5 years are at greatest risk of illness and death [1, 2]. Rapid diagnostic tests (RDTs) reliably diagnose malaria at the point of care and identify children to be treated with artemisinin-based combination therapy (ACT); rectal artesunate is recommended for patients who can no longer take oral drugs before transfer to a facility for continued management [3]. Data on the feasibility of providing an integrated package are essential, particularly in remote rural areas where malaria has not yet declined [3–5]. Evidence is needed about the potential health benefits as well as costs and savings of introducing such interventions in underserved areas.

Malaria can be successfully treated with existing medicines, but access to them is limited in affected populations. In rural Africa, households frequently rely on private, for-profit providers where they make direct payments for healthcare, due to long distances to public facilities, or because such facilities do not function adequately or do not have appropriate medicine and consumable stocks [6–8]. The burden of paying privately for healthcare is a major financial cost to the household, and is exacerbated by income lost from not being able to carry out normal activities because malaria often occurs in the rainy season, simultaneous with an increased need for labor on the farm. Ill health and the associated costs tip families into catastrophic economic conditions, recovery from which may take considerable time [9, 10]. At the same time, there is an increasing literature on interventions using community health workers (CHWs) to successfully provide basic care [11–13]. A number of interventions have demonstrated the extent to which CHWs can substantially improve health when trained adequately and when provided with commodities and supervision [14, 15]. CHWs have been shown to be able to assess and diagnose children and improve access to healthcare by reaching the poorest in the most inaccessible areas. Several studies have shown a reduction in child mortality associated with CHWs’ provision of care [13–16].

As part of an implementation study that trained CHWs to assess sick children, diagnose malaria using RDTs, and treat with ACTs or rectal artesunate in 3 African countries (Burkina Faso, Nigeria, and Uganda) chosen because they are among the top 10 countries contributing 80% of global malaria cases [17], we studied the private household costs for a febrile episode in a child before the intervention was launched, and after the CHWs’ training was completed and the intervention was in force. Our objective was to quantify the impact of this intervention improving access to malaria diagnosis and treatment on private household costs of illness, and to provide policy makers with reliable estimates of these costs if they were to consider scaling up the intervention at the district or national level.

## METHODS

### Study Sites

The main intervention study was carried out in 3 rural areas of Burkina Faso (health area of Sidéradougou, Health District of Mangodara), Nigeria (Ona-Ara local government area), and Uganda (Sheema district) in 2014, and consequently the pre-intervention household questionnaire was implemented between May and July 2013 (Nigeria and Uganda) and in July 2014 (Burkina Faso) to understand how people normally managed febrile illness (mostly malaria) before the intervention, and to quantify the costs they incurred.

In total, 45, 33, and 32 study communities were included in Burkina Faso, Nigeria, and Uganda, respectively, during the pre-intervention phase and 45, 33, and 84 communities during the intervention; in Uganda, Kayunga district was added to the intervention study. ACTs and rectal artesunate were provided to all CHWs, except in Burkina Faso, where rectal artesunate was provided to 31 out of 50 CHWs.

In all study areas, households are mainly agricultural, and therefore, dependent upon the rains for irrigating crops. The malaria season is also associated with rainfall, because the rains increase the number of mosquitoes that can transmit *Plasmodium falciparum* parasites to humans, mainly nonimmune children. In Mangodara, Burkina Faso, malaria transmission peaks between June to August and sometimes tails off in November/December, and in the Ona-Ara area, Nigeria, malaria peaks between May and October. However, in Uganda, malaria is seasonal in Sheema district (between March and June/July and September/October) but stable through the whole year in Kayunga district.

### Health Provision in the Study Area

In all 3 countries, people typically go for advice and care for suspected malaria to traditional healers, faith homes (churches), CHWs, traditional birth attendants (Nigeria), drug shops/patent medicine sellers, drug hawkers, maternities, dispensaries (not in Nigeria), health centers, or private clinics (Nigeria and Uganda). The closest hospital is situated 25 km away from the study area in Burkina Faso, approximately 8 km (private hospital) or between 20 and 22 km (public hospital) away in Nigeria, and 36 km away for Sheema district (Uganda); in Kayunga district (Uganda), it takes about 2–3 hours walking from Kayunga's Grade IV health center to the hospital.

In Burkina Faso, healthcare at any public facility is fee-based. Consultation costs about 200 West African CFA francs (XOF; US Dollars [USD] $.33) and when admitted, patients pay for their bed. In Nigeria and Uganda, consultations, drugs, and bed costs at public facilities are theoretically provided free of charge for children <5 years old, but caregivers still have to pay for a registration card and injections (Nigeria) or an exercise book and drugs bought outside the hospital (Uganda). In all 3 countries, shops and pharmacies sell quinine, antibiotics, and antimalarials without prescription.

Before the intervention, no RDTs, ACTs, or rectal artesunate were available to the communities in the public sector. CHWs were available but relatively dormant as a means of providing healthcare. During the intervention, RDTs and drugs were provided free of charge in the study areas of Nigeria and Uganda. However, in Burkina Faso, RDTs and rectal artesunate were free of charge, but ACT was sold (as per public policy) to CHWs at a cost of 70 XOF (USD $.12; 6 tablets per blister pack) for children aged <37 months and 150 XOF (USD $.25; 12 tablets per blister pack) for children aged 37–59 months. Each CHW was authorized to make a nominal profit by charging parents these costs plus a nominal profit of 30 XOF (USD $.05) for each young child treated with 6 ACTs and 50 XOF (USD $.08) for each older child treated with 12 ACTs.

### Household Questionnaires on Costs

The purpose of the study questionnaire was to determine treatment-seeking behavior and expenditure for a recent childhood illness by households randomly selected from the villages before and during the intervention. In all 3 countries, a visit was made to households, and if they had a child who was between 6 and 59 months of age and had a fever in the preceding 2 weeks, questions were asked regarding the illness, healthcare received, and their healthcare expenses. Households that did not have children who were ill, or whose caregiver was not present during the illness or who refused to give consent, were excluded from interview.

### Questionnaire Content, Selection of the Interviewers, and Data Collection

Two questionnaires (one for each phase) were developed: in French for Burkina Faso and in English for Nigeria and Uganda. In Nigeria and Uganda, these questionnaires (hereinafter called case report forms [CRFs]) were translated into local languages (Yoruba in Nigeria, and Luganda in Kayunga district and Lunyankole in Sheema district, Uganda) and pilot tested for comprehension before use. For the pre-intervention phase, there were 10 interviewers in Burkina Faso and Nigeria and 8 in Uganda; for the intervention phase, there were 10 in Burkina Faso, 13 in Nigeria, and 20 in Uganda. Interviewers were fluent in local languages and had good education and prior experience in research/data collection. Most interviewers were used for both pre-intervention and the intervention phase. In all 3 countries, interviewers were trained for at least 1 day. Training sessions were interactive with question-and-answer sessions and role plays. Each interviewer was tested through completing CRFs before certification.

Each interview lasted between 20 minutes and 1 hour. Although the pre-intervention and intervention CRFs were adapted to the country, they contained the same main questions on general sociodemographic context of the household, clinical course of the episode (timing, symptoms, actions taken, healthcare providers visited), costs incurred (for transportation, medicines, registration/consultation fees, laboratory/diagnostic tests, accommodation, and food for each consultation) and what happened during a consultation visit to a health provider (RDTs, treatments). The CRF during intervention was identical to the pre-intervention CRF but attempted to capture additional information on reasons for not going to a CHW where relevant.

### Sampling Strategy

In calculating our sample size for household surveys, we assumed that the average number of children <5 years of age per household was 2, except in Uganda, where the average was assumed as 1.4. Requiring a precision of ±5% points for the point estimates of proportions, a minimum sample size would be 384 for the worst-case scenario of 50%. Accounting for a design effect of 2 for clustering on household level would give us a total sample size of 768 interviews before and during the intervention in each country. We achieved interviews with 1856 caretakers in Burkina Faso (514 before, 1342 during), 1560 in Nigeria (775 before, 785 during), and 1529 in Uganda (457 before and 1072 during), with a total of 4945 households interviewed.

### Data Analysis and Statistical Methods

#### Baseline Characteristics

Demographic information on the child and household (gender, age, education, occupation, food problems, number of working people in the household) and information on the illness episode (danger signs, number of children who went to a CHW/trained health worker, main reasons for not going to a CHW, number of days lost) was obtained. Baseline characteristics for sampled illness cases were compared before and during intervention.

#### Private Costs

Household costs reported by the parent or guardian of the child were categorized into registration, consultation, user fee, diagnosis, drugs, bed, food, informal, transport, and other costs for caretaker or patient. Transport cost included costs of the parent or guardian but excluded costs paid by a third party (ie, a person not related to the household) accompanying the parent/guardian and child. In addition, the costs reported by caregivers of children who were still sick at the time of interview were excluded from the analysis as it would underestimate the total cost for a whole episode of illness. Total private costs per illness episode before and during intervention were compared separately for each country. Costs during the intervention were also compared between those who went to a CHW and those who did not. In the latter comparison, total costs were further stratified by severity of the episode (uncomplicated vs severe). Costs are presented in USD using the average exchange rate in the period May 2013 to August 2014: 1 USD = 487.80 XOF (Burkina Faso); 1 USD = 161.76 Nigerian naira (NGN); 1 USD = 2631.58 Ugandan shilling (UGX) for the pre-intervention phase; and the average exchange rate between April and October 2015: 1 USD = 598.09 XOF; 1 USD = 200.36 NGN; 1 USD = 3472.22 UGX for the intervention phase (www.oanda.com).

#### Projecting Impact of Intervention on Household Costs at the Whole District Level

To project the impact of intervention on household costs at district level, external data were used for the number of children <5 years old in each district and the number of malaria episodes per year and per child <5 years old. Estimates of the impact per child and per episode are presented and mean cost savings were then calculated for malaria episodes per year. Overall cost impacts were converted into USD.

#### Statistical Methods

For each phase and each country separately, all data were double entered in EpiData 3.1 and analyzed using Stata software, version 13.0 (StataCorp, College Station, Texas). A Student *t* test was performed on the equality of means with a level of significance of *P* = .05 and a confidence level of 95%, and a test for heterogeneity was used to compare baseline characteristics of participating households before and during the intervention by country and overall.

### Ethical Considerations

The research protocol of the main study was approved by the National Health Research Committee, the University of Ibadan/University College Hospital Institutional Review Committee, and Oyo State Ministry of Health in Nigeria; the National Ethics Committee for the Research on Health and the National Regulatory Authority in Burkina Faso; the National Council for Science and Technology in Uganda; and the World Health Organization Research Ethics Review Committee. In addition, Nigeria obtained permission from the local government secretariat as well as from the head of the communities. In Burkina Faso, additional approvals were obtained from the community via their leader by signing a written consent, and in Uganda, from the district heath officer and the local council I (village level). Finally, in Burkina Faso as well as in Uganda, oral consent was obtained from caregivers while written consent was obtained in Nigeria.

## RESULTS

### Baseline Characteristics

The majority of children in the study were <36 months of age (60% before and 63% during intervention) (Table 1). Respondents were mainly female (80% before and 96% during intervention), most being <36 years old (69% before and 77% during intervention). In Burkina Faso, most of the caregivers had never been to school (80% before and 89% during intervention) while in Nigeria and Uganda, the caretakers were better educated and many had completed primary school (Nigeria: 40% before and 35% during intervention; Uganda: 62% before and 61% during intervention). Income was mainly based on agriculture in Burkina Faso and Uganda, and most of the households declared they did not have food problems (Burkina Faso: 70% before and 74% during intervention; Uganda: 54% before and 44% during intervention), while in Nigeria, households sometimes had food problems (30% before and 43% during intervention). In Nigeria, a large proportion of caregivers were self-employed (57% before and 71% during intervention). Most episodes were without danger signs (75% before vs 82% during intervention). Before the intervention, 35% of caretakers went to a CHW while 50% went during the intervention (*P* < .0001). For those who did not go to a CHW during the intervention, the main reasons were that parents believed that their child was not very sick (18%), were not aware of the presence of the CHW in their community (13%), believed that CHWs cannot treat well (8%), or because the CHW was often not at home (7%) (Supplementary Table 1).

**Table 1.** Characteristics of Children, Caregivers, Caregivers' Households, and Childhood Illness in the Study

Category	Before Intervention								During Intervention							
	Burkina Faso		Nigeria		Uganda		Total		Burkina Faso		Nigeria		Uganda		Total	
	No.	%	No.	%	No.	%	No.	%	No.	%	No.	%	No.	%	No.	%
Total No. of children/cases	514	29	775	45	457	26	1746	100	1342	42	785	24	1072	34	3199	100
Child characteristics																
Child's age																
<36 mo	327	64	448	58	282	62	1057	60	905	67	439	56	687	64	2031	63^a^
≥36 mo	178	34	326	42	175	38	679	39	437	33	346	44	367	34	1150	36^a^
Caregiver and household characteristics																
Caregiver's gender																
Male	220	43	62	8	64	14	346	20	29	2^a^	17	2^a^	77	7^a^	123	4^a^
Female	294	57	713	92	393	86	1400	80	1313	98^a^	768	98^a^	992	93^a^	3073	96^a^
Caregiver's age, y																
15–24	82	16	123	16	92	20	297	17	325	24^a^	130	17	277	26^a^	732	23^a^
25–35	225	44	461	59	226	50	912	52	786	59^a^	452	58	498	47^a^	1736	54^a^
36–50	153	30	137	18	100	22	390	22	182	13^a^	168	21	226	21^a^	576	18^a^
>50	34	7	32	4	37	8	103	6	11	1^a^	32	4	47	4^a^	90	3^a^
Education																
No education	408	80	189	25	55	12	652	37	1202	89^a^	171	22^a^	120	11	1493	47^a^
≤7 y	43	8	312	40	281	62	636	36	106	8^a^	279	35^a^	652	61	1037	32^a^
>7 y	21	4	273	35	121	26	415	24	26	2^a^	335	43^a^	280	26	641	20^a^
Occupation																
Unemployed	11	2	27	4	9	2	47	3	35	3^a^	1	0^a^	31	3	67	2^a^
Agriculture only	470	92	270	35	370	81	1110	63	687	51^a^	190	24^a^	794	74	1671	52^a^
Employed (only or + agriculture)	2	0	27	4	17	4	46	3	10	1^a^	40	5^a^	45	4	95	3^a^
Self-employed (only or + agriculture)	30	6	415	53	59	13	504	29	610	45^a^	465	59^a^	186	18	1261	40^a^
Others^b^	…	…	34	4	2	0	36	2	…	…	89	12^a^	3	0	92	3^a^
Food problems																
Never	357	70	188	24	246	54	791	45	994	74^a^	165	21^a^	470	44^a^	1629	51^a^
Seldom	57	11	300	39	91	20	448	26	201	15^a^	208	26^a^	182	17^a^	591	18^a^
Sometimes	57	11	237	30	79	17	373	21	108	8^a^	337	43^a^	261	24^a^	706	22^a^
Often	25	5	37	5	27	6	89	5	33	3^a^	61	8^a^	86	8^a^	180	6^a^
Always	2	0	…	…	14	3	16	1	2	0^a^	14	2^a^	45	4^a^	61	2^a^
No. of working people over 10 y of age in household																
≤2	41	8	656	85	397	87	1094	62	603	45^a^	696	89^a^	883	82	2182	68^a^
3–5	176	34	116	15	54	12	346	20	663	50^a^	79	10^a^	161	15	903	28^a^
6–10	198	39	3	0	6	1	207	12	73	5^a^	9	1^a^	27	3	109	4^a^
>10	88	17	…	…	…	…	88	5	3	0^a^	1	0^a^	…	…	4	0^a^
Illness characteristics																
Danger signs																
No	437	85	588	76	290	63	1315	75	1129	84	631	80^a^	859	80^a^	2619	82^a^
Yes	77	15	187	24	167	37	431	25	213	16	154	20^a^	213	20^a^	580	18^a^
No. going to trained CHWs^c^																
Trained CHWs/health worker^d^	105	20	515	66^d^	…	…	620	35	603	45^a^	534	68	469	44^a^	1606	50^a^
Other providers	409	80	260	34	457	100	1126	65	739	55^a^	251	32	603	56^a^	1593	50^a^

Missing data: child's age: 10 before and 18 during; caregiver's gender: 3 during; caregiver's age: 44 before and 65 during; education: 43 before and 28 during; occupation: 3 before and 13 during; food problems: 29 before and 32 during; number of working people >10 years of age in household: 11 before and 1 during.

Abbreviation: CHW, community health worker.

^a^ Test for heterogeneity: during intervention vs before intervention: *P* < .05.

^b^ Combination of agriculture, employed, and self-employed.

^c^ In Nigeria, CHWs as well as shop owners were trained as part of the intervention.

^d^ In Nigeria, pre-intervention data included patients who went to a health center, hospital, or maternity center. None went to a CHW during this period.

### Household Out-of-Pocket Costs for Illness Episode

Table 2 presents the out-of-pocket costs incurred by caregivers of sick children before and during the intervention split into different cost categories. During the intervention, total household costs per episode were lower in all 3 countries, about 3 times lower in Burkina Faso: USD $1.54 vs USD $4.36 (difference, USD $2.82 [95% confidence interval {CI}, USD $2.09–USD $3.55]; *P* < .0001), twice lower in Nigeria: USD $2.04 vs USD $3.90 (difference, USD $1.86 [95% CI, USD $1.01–USD $2.70]; *P* < .0001), and 3 times lower in Uganda: USD $1.42 vs USD $4.46 (difference, USD $3.04 [95% CI, USD $2.29–USD $3.79]; *P* < .0001). Similar decreases were observed for total costs for those who incurred some costs, with the largest reductions observed in drug and transport costs (drugs: USD $1.74 during vs USD $4.15 before intervention; transport: USD $1.42 vs USD $2.49, respectively). The percentage of households that paid something during the episode decreased for both Nigeria (12% less) and Uganda (15% less) but slightly increased for Burkina Faso (+2%). Comparing those who went to a CHW during the intervention with those who did not, those who went had lower costs: USD $1.32 vs USD $2.03 (difference, USD $.71 [95% CI, USD $.34–USD $1.09]; *P* < .0001) (Supplementary Table 2), and more than half of them had a RDT (94% in Burkina Faso, 55% in Nigeria, and 66% in Uganda; unpublished data). There was no difference in time spent by the guardian in caring for the sick child (59% before vs 51% during intervention spent ≤2 days; Table 2).

**Table 2.** Mean Private Costs (in US Dollars) per Episode of Illness Before and During Intervention**^a^**

	Before Intervention								During Intervention							
	Burkina Faso		Nigeria		Uganda		Total		Burkina Faso		Nigeria		Uganda		Total	
Category	No.	Mean (SD)	No.	Mean (SD)	No.	Mean (SD)	No.	Mean (SD)	No.	Mean (SD)	No.	Mean (SD)	No.	Mean (SD)	No.	Mean (SD)
Costs (in USD)^a^																
Registration	26	0.65 (0.9)	26	1.69 (1.3)	1	0.08 (…)	53	1.15 (1.2)	…	…	7	1.69 (1.8)	1	1.50 (…)	8	1.66 (1.6)
Consultation	101	0.51 (0.8)	4	4.15 (2.2)	1	5.81 (…)	106	0.69 (1.2)	184	0.35 (0.1)	5	3.79 (2.4)	1	1.50 (…)	190	0.44 (0.7)
User fee	1	1.03 (…)	…	…	…	…	1	1.03 (…)	…	…	…	…	4	1.75 (1.8)	4	1.75 (1.8)
Diagnosis	3	8.68 (11.5)	10	3.20 (2.7)	32	1.10 (1.0)	45	2.07 (3.5)	3	2.65 (2.9)	6	1.95 (1.1)	22	1.02 (0.6)	31	1.36 (1.1)
Drugs^b^	297	3.17 (5.2)	568	3.77 (6.1)	233	6.35 (9.6)	1098	4.15 (6.9)	913	1.23 (2.7)	495	2.41 (6.7)	273	2.25 (4.0)	1681	1.74 (4.5)
Bed	4	2.31 (1.0)	3	32.92 (30.7)	7	2.31 (1.9)	14	8.87 (17.8)	7	2.47 (2.0)	…	…	2	2.68 (0.4)	9	2.52 (1.7)
Food	45	1.16 (1.7)	2	1.14 (1.0)	54	1.23 (2.1)	101	1.20 (1.9)	55	1.03 (1.4)	13	1.60 (2.7)	151	1.18 (1.8)	219	1.17 (1.8)
Informal	…	…	…	…	1	0.19 (…)	1	0.19 (…)	1	0.51 (…)	…	…	4	0.45 (0.7)	5	0.46 (0.6)
Transport	120	3.41 (8.0)	103	1.87 (1.4)	98	2.03 (1.5)	321	2.49 (5.1)	226	1.52 (0.9)	47	1.25 (1.3)	82	1.25 (1.2)	355	1.42 (1.1)
Other	…	…	…	…	1	0.58 (…)	1	0.58 (…)	8	1.55 (1.9)	3	3.33 (1.4)	2	0.40 (0.1)	13	1.78 (1.9)
Total costs for those who paid something	302	4.99 (10.9)	578	4.37 (9.2)	283	6.37 (9.8)	1163	5.02 (9.8)	934	1.74 (3.8)*	498	2.66 (7.4)**	364	2.57 (4.5)*	1796	2.16 (5.2)*
Total costs for all completed episodes^c^	346	4.36 (10.3)	648	3.90 (8.8)	404	4.46 (8.7)	1398	4.18 (9.2)	1056	1.54 (3.6)*	650	2.04 (6.6)*	658	1.42 (3.6)*	2364	1.64 (4.6)*
Total costs, uncomplicated episodes	293	4.31 (10.7)	492	3.60 (6.7)	258	4.49 (7.4)	1043	4.02 (8.2)	905	1.41 (3.4)*	529	1.87 (6.0)*	525	1.35 (3.4)*	1959	1.52 (4.3)*
Total costs, severe episodes	53	4.62 (7.8)	156	4.84 (13.5)	146	4.42 (10.5)	355	4.64 (11.6)	151	2.32 (4.5)***	121	2.78 (8.7)	133	1.73 (4.2)***	405	2.26 (6.0)**
No. of children still sick at interview^d^	168	…	127	…	53	…	348	…	280	…	135	…	411	…	826	…
Households that incurred cost	87%	…	89%	…	70%	…	83%	…	89%	…	77%	…	55%	…	76%	…
Difference in households paying: intervention vs pre-intervention	…	…	…	…	…	…	…	…	2%	…	−12%	…	−15%	…	−7%	…
	No.	%	No.	%	No.	%	No.	%	No.	%	No.	%	No.	%	No.	%
No. days lost caring for sick children: completed episodes^e^																
No/little loss of time	…	…	…	…	2	1	2	0	…	…	…	…	…	…	…	…
≤2 d	215	62	448	69	159	39	822	59	380	36	515	79	306	46	1201	51
>2–5 d	112	32	153	24	175	43	440	32	643	61	114	17	272	41	1029	44
>5–8 d	12	4	26	4	56	14	94	7	19	2	17	3	63	10	99	4
>8 d	2	1	5	1	12	3	19	1	3	0	4	1	6	1	13	0
Missing /unknown	5	1	16	2	…	…	21	1	11	1	…	…	11	2	22	1

Abbreviations: SD, standard deviation; USD, US dollars.

^a^ Mean costs are presented for completed episodes. Data on children who were still sick at the time of the interview were excluded as their episode costs were incomplete. Numbers of children still sick at the time of interview and percentage of households who paid something for the illness are provided in the table.

^b^ Nigeria: Artemisinin-based combination therapies were normally provided at no cost by drug shop owners trained for the study. However, when someone stated they went to a drug shop, they did not know whether the person was trained or not.

^c^ Two hundred thirty-five patients in the pre-intervention period and 568 patients in the intervention period did not incur any costs for a completed episode.

^d^ Intervention: Outcome missing for 6 children in Burkina Faso and 3 children in Uganda.

^e^ Test for heterogeneity in distributions during intervention vs before intervention: *P* < .0001 for Burkina Faso, *P* = .0166 for Nigeria, *P* = .0039 for Uganda, *P* < .0001 for total.

*P < .0001; **P < .001; ***P < .01.

### Cost Savings Due to the Intervention at Household and District Levels

Table 3 presents estimates of the total household costs saved per year due to the intervention. With 1.18 (Burkina Faso [18]), 3.5 (Nigeria [19]), and 0.98 (Uganda [20]) malaria episodes per year per child and a private household cost savings per episode of USD $2.82 (Burkina Faso), USD $1.86 (Nigeria), and USD $3.04 (Uganda), the projected total mean household cost savings at district level per year were USD $29 965 for Mangodara (Burkina Faso), USD $254 268 for Ona-Ara (Nigeria), and USD $303 467 for Sheema and Kayunga (Uganda).

**Table 3.** Estimated Household Savings (in US Dollars) for Malaria Due to the Intervention for the Whole District of the Study Areas

Category	Mangodara, Burkina Faso	Ona-Ara, Nigeria	Sheema and Kayunga, Uganda
No. of malaria episodes per year, per child <5 y old^a^	1.18	3.50	0.98
No. of children <5 y old^b,c^	9005	39 058	101 862
Mean household costs (USD) per episode (SD)^d^ before intervention	4.36 (10.3)	3.90 (8.8)	4.46 (8.7)
Mean household costs (USD) per episode (SD)^d^ during intervention	1.54 (3.6)	2.04 (6.6)	1.42 (3.6)
Savings per child (USD) per episode (SE)^e^	2.82 (0.6)	1.86 (0.4)	3.04 (0.5)
Total mean savings (USD) per child and per year (SE)^e^	3.33 (0.7)	6.51 (1.5)	2.98 (0.5)
Before intervention: total mean costs (USD) per year for malaria for the whole district	46 329	533 142	445 218
During intervention: total mean costs (USD) per year for malaria for the whole district	16 364	278 874	141 751
Total mean savings (USD) per year for malaria for the whole district	29 965	254 268	303 467

Abbreviations: SD, standard deviation; SE, standard error; USD, US dollars.

^a^ References [18–20]

^b^ References [21–23].

^c^ For Mangodara, Sheema, and Kayunga: To calculate the number of children <5 years of age in the district, we used the total population in the district and the percentage of children <5 years in the country (based on the total number of children <5 years and the total population in the country).

^d^ Mean costs (SD) per completed episode.

^e^ The number of malaria episodes/child was assumed to be fixed (ie, without uncertainty).

## DISCUSSION

In this study in 3 malaria-endemic African countries, we quantified household costs incurred in managing an episode of febrile illness before and after integrated provision of RDTs, ACTs, and rectal artesunate by CHWs. There was a >2-fold reduction in household private costs per illness episode as a consequence of the intervention—from USD $4.18 before to USD $1.64 during the intervention. The findings were separately significant for each country, although the magnitude was larger in Burkina Faso and Uganda (two-thirds reduction) than in Nigeria (reduction of approximately 40%). Cost categories with largest reductions were drug costs (reducing from USD $4.15 to USD $1.74 for those reporting such costs) and transport (from USD $2.49 to USD $1.42, respectively).

CHWs were located closer to patients; consequently, caregivers were more likely to seek care faster, and we expected this to halt the progression of the disease and decrease the length of the illness episode. We found evidence of this through a significant reduction in the proportion of children with danger signs requiring referral to hospital during the intervention: 24.7% vs 18.1% [17]. The illness recovery was shorter during the intervention, reducing from 3.69 to 3.47 days [17]. Those who went to a CHW during the intervention incurred significantly lower out-of-pocket costs compared with those who did not do so. Household private costs per episode during the intervention for those who went to a CHW was approximately 63% lower in Burkina Faso and approximately 44% lower in Uganda, but remained similar in Nigeria, where drug costs were substantial even during the intervention.

Mean household savings per child per year due to the intervention constitutes an important component of household budgets as the money saved through more rapid access to diagnosis and treatment can be used for other household needs. On a district basis, the cost savings would be expected to be considerable, depending on the number of children who become ill (illness frequency was less but the child population was greater in the study areas of Uganda compared with the study areas of Burkina Faso and Nigeria), making the cost reduction substantial in all 4 study districts.

CHWs were located closer to patients and were provided either with free (or low-cost) drugs and diagnostics; both of these features of the intervention may have changed caregivers’ behavior. A parallel qualitative study on the acceptability of the intervention indicated that during the intervention caregivers stated that they were more likely to choose the CHW because they were aware that CHWs had diagnostics and free drugs and they were more likely to go where there were commodities instead of seeking other alternatives [24]. Second, there is evidence that early treatment of uncomplicated malaria reduces the risk of severe malaria and hence hospital referral and associated transport costs [8]. Nevertheless, some caretakers did not go to a CHW during the intervention (55% in Burkina Faso, 32% in Nigeria, and 56% in Uganda). The main reasons were a belief that their child was not sufficiently sick, not being aware of the CHW presence in their community, or the CHW not being at home because of farm duties. Many participants in Uganda also mentioned stockout of drugs at the CHW during the intervention [24]. To ensure better use of CHWs and access to care at the community level, future implementations should aim to better sensitize communities, provide advance information of the presence of the CHWs and symptoms of malaria, and improve stock management. Compensation for CHWs’ time might be explored as motivation for CHWs to be more available when needed.

The Global Fund from which most malaria-endemic countries apply for commodities has, during the past 5 years, indicated that essential items for malaria control can be secured through successful applications; these include training and salary costs for community health workers, RDTs for malaria diagnosis, ACTs for malaria treatment, supportive supervision, supply chain system strengthening, and health information system strengthening (http://www.rollbackmalaria.org/files/files/resources/HWG-2014-country-briefing-note.pdf). The Global Fund still continues to encourage applicants to include community system strengthening initiatives in proposals wherever relevant to improve health outcomes (www.theglobalfund.org/documents/core/infonotes/Core_CSS_InfoNote_en/). Consequently, our results provide governments with an understanding of how the system can work in their countries in support of the role advocated by the Global Fund and provide a strong economic rationale for scaling up the intervention in underserved areas to improve the speed of malaria diagnosis and treatment. The main barriers that can be anticipated, as identified in this study, are underuse of CHWs because of a lack of awareness, financial constraints (mainly transport to a CHW), beliefs about the etiology of disease, and limited autonomy of caregivers.

Our study has several limitations. Perhaps the most important is that data on costs are for all febrile illnesses in children both before and during the intervention. Few RDTs were used before the intervention, and therefore, costs before the intervention would necessarily include any febrile illness. Consequently, the cost reduction could not exclude costs of managing nonmalaria illnesses. However, during the study, the vast majority of cases seen by CHWs were malaria-positive (81.2%), and there were no differences in household costs per episode between malaria-positive and malaria-negative cases overall and separately in the 3 participating countries. Second, we kept cost calculations to documented costs provided by the caretaker for the episode of the child. We did not calculate the lost income due to time taken to look after the sick child. Third, in Burkina Faso, in the period before intervention, for cultural reasons, fathers of sick children answered the questionnaire as would be culturally normal in the district; during the intervention period, the study team explained that the primary caretaker, usually the mother, should answer questions. In the localities in which the study was conducted, it was probable that both parties might be present during the interview, but it is not possible to rule out bias in the answers provided on costs. Finally, all costs were based on interviews and were therefore dependent upon participants’ recollection of costs incurred within the 2 weeks prior to interview.

## Supplementary Data

Supplementary materials are available at http://cid.oxfordjournals.org. Consisting of data provided by the author to benefit the reader, the posted materials are not copyedited and are the sole responsibility of the author, so questions or comments should be addressed to the author.

Supplementary Data
