# Supplementary material for: Impact of Improving Community-Based Access to Malaria Diagnosis and Treatment on Household Costs
Source: Clin Infect Dis. 2016 Dec 6;63(Suppl 5):S256–63. doi: 10.1093/cid/ciw623 (PMC5146695; doi:10.1093/cid/ciw623)
Supplement: Supplementary Data [file supp_63_suppl-5_S256__index.html]

Supplementary Data 

# Impact of Improving Community-Based Access to Malaria Diagnosis and Treatment on Household Costs

## Supplementary Data

Supplementary Data

- Supplementary Data - Pdf file
