# Supplementary material for: Impact of Improving Community-Based Access to Malaria Diagnosis and Treatment on Household Costs
Source: Clin Infect Dis. 2016 Dec 6;63(Suppl 5):S256–63. doi: 10.1093/cid/ciw623 (PMC5146695; doi:10.1093/cid/ciw623)
Supplement: Supplementary Data [file supp_ciw623_ciw623supp.pdf]

Supplementary Table 1. Reasons for not going to a CHW during the intervention

Main reasons for not going to a CHW*	Burkina Faso		Nigeria		Uganda		Total	
	N	%	N	%	N	%	N	%
**Number of respondents who did not go to a trained CHW/health worker****	**739**	**-**	**251**	**-**	**603**	**-**	**1593**	**-**
Not aware of the presence of CHW	78	11	6	2	120	20	204	13
CHW lives too far away	48	6	-	-	39	6	87	5
CHW often not at home	36	5	1	0	73	12	110	7
Afraid that cannot pay	10	1	7	3	3	0	20	1
Child was not too sick	232	31	21	8	30	5	283	18
CHW cannot treat well	92	12	1	0	28	5	121	8
Not satisfied with previous treatment	1	0	-	-	9	1	10	1
Prefer traditional medicine for those symptoms	73	10	2	1	5	1	80	5
Don’t like the drugs given by CHW	2	0	-	-	7	1	9	1
I was too busy or sick	10	1	9	4	6	1	25	2
Not allowed from husband/elders	16	2	0	0	6	1	22	1
Others	301	41	38	15	306	51	645	40
Missing	9	1	178	71	35	6	222	13

* A multiple choice question. Most of the participants in Nigeria did not answer this question as this question was added at a later stage.

** In Nigeria CHWs as well as shop owners were trained as part of the intervention.

Supplementary Table 2. Mean private costs (in USD) per episode of illness for those who went to a CHW vs. those who did not go to a CHW during the intervention[Ω]

Category	NO VISIT TO A CHW<br>Burkina Faso (N=576)	Nigeria (N=185)*	Uganda (N=318)	Total (N=1079)	VISIT TO A CHW<br>Burkina Faso (N=480)	Nigeria (N=465)*	Uganda (N=340)	Total (N=1285)
	**Mean (SD)**	**Mean (SD)**	**Mean (SD)**	**Mean (SD)**	**Mean (SD)**	**Mean (SD)**	**Mean (SD)**	**Mean (SD)**
**Costs (in USD)**								
Registration	-	0.06 (0.45)	0.005 (0.08)	0.01 (0.19)	-	0.001 (0.02)	-	0.0005 (0.01)
Consultation	0.10 (0.16)	0.06 (0.60)	0.005 (0.08)	0.06 (0.28)	0.01 (0.08)	0.02 (0.25)	-	0.01 (0.16)
User Fee	-	-	0.002 (0.03)	0.0006 (0.02)	-	-	0.02 (0.25)	0.005 (0.13)
Diagnosis	0.01 (0.26)	0.06 (0.39)	0.06 (0.27)	0.03 (0.29)	-	0.0005 (0.01)	0.01 (0.13)	0.004 (0.07)
Drugs**	1.53 (3.00)	1.69 (2.44)	1.26 (2.51)	1.48 (2.77)	0.50 (1.59)	1.90 (6.89)	0.63 (2.97)	1.04 (4.57)
Bed	0.01 (0.16)	-	-	0.007 (0.12)	0.02 (0.33)	-	0.02 (0.21)	0.01 (0.23)
Food	0.07 (0.46)	0.01 (0.11)	0.31 (1.01)	0.13 (0.65)	0.04 (0.30)	0.04 (0.50)	0.24 (1.02)	0.09 (0.64)
Informal	-	-	0.005 (0.08)	0.002 (0.05)	0.001 (0.02)	-	0.0002 (0.003)	0.0004 (0.01)
Transport	0.42 (0.86)	0.05 (0.26)	0.21 (0.70)	0.29 (0.75)	0.22 (0.60)	0.11 (0.54)	0.10 (0.47)	0.15 (0.55)
Other	0.01 (0.13)	0.05 (0.45)	0.0009 (0.02)	0.01 (0.21)	0.02 (0.27)	-	0.001 (0.03)	0.006 (0.17)
**TOTAL COSTS******	2.15 (4.32)	1.99 (3.02)	1.85 (3.55)	2.03 (3.90)	0.80 (2.36)	2.06 (7.53)	1.03 (3.53)	1.32 (5.11)
**P value: No CHW vs. CHW**	-	-	-	-	**p<0.0001**	p=0.5486	**p=0.0015**	**p=0.0001**

	**N**	**Mean (SD)**	**N**	**Mean (SD)**	**N**	**Mean (SD)**	**N**	**Mean (SD)**	**N**	**Mean (SD)**	**N**	**Mean (SD)**	**N**	**Mean (SD)**	**N**	**Mean (SD)**
**TOTAL COSTS Uncomplicated episode**	504	1.99 (4.3)	154	1.82 (2.9)	238	1.69 (2.9)	896	1.88 (3.8)	401	0.68 (1.6)	375	1.89 (6.8)	287	1.06 (3.7)	1063	1.21 (4.6)
**P value: Not CHW vs. CHW**	-	-	-	-	-	-	-	-	-	**p<0.0001**	-	p=0.5496	-	**p=0.0160**	-	**p=0.0002**
**TOTAL COSTS Severe episode**	72	3.28 (4.3)	31	2.83 (3.4)	80	2.30 (5.0)	183	2.78 (4.5)	79	1.46 (4.5)	90	2.76 (9.9)	53	0.86 (2.2)	222	1.84 (7.0)
**P value: Not CHW vs. CHW**	-	-	-	-	-	-	-	-	-	**p=0.0062**	-	p=0.4844	-	**p=0.0256**	-	p=0.0599

Ω Mean costs are presented for completed episodes. Data on children who were still sick at the time of the interview were excluded as their episode costs were incomplete.

* In Nigeria: costs of those who went to a drug shop and hospital workers were included in the "visit to a CHW" section as shop owners were trained as part of the intervention and hospital workers may have RDTs with them.

** Nigeria: ACTs were normally provided at no cost by drug shop owners trained for the study. However, when someone stated they went to a drug shop, they did not know whether the person was trained or not.

*** Total costs for uncomplicated and severe episodes: Burkina Faso: total costs decreased by 62.79% (1-0.80/2.15); Nigeria: total costs increased by 3.40% (1-1.99/2.06); Uganda: total costs decreased by 44.32% (1-1.03/1.85).
